# Survival of Rheumatic Heart Disease in Indonesian Children

**DOI:** 10.5334/gh.1160

**Published:** 2022-10-06

**Authors:** Nadya Arafuri, Indah Kartika Murni, Madarina Julia, Sasmito Nugroho, Noormanto Soehadi

**Affiliations:** 1 Department of Child Health, Faculty of Medicine, Public Health and Nursing, Universitas Gadjah Mada, Dr Sardjito Hospital, Yogyakarta, ID

**Keywords:** Rheumatic heart disease, Survival rates, Children, Mortality

## Abstract

**Background:**

Rheumatic heart disease (RHD) remains a preventable cause of cardiovascular deaths in children in low- and middle-income countries. However, long-term outcome data of children with RHD is still lacking in Indonesia.

**Objective:**

To investigate the long-term outcomes of RHD, particularly the survival rates and the predictors.

**Methods:**

A retrospective cohort study was done in children aged less than 18 years old admitted with RHD at a tertiary hospital (Dr. Sardjito Hospital, Indonesia) from 2011–2021. Survival time was estimated from the date of first diagnosis of RHD to the survival endpoint (date of mortality or censoring). Kaplan-Meier curves, log-rank test and Cox-regression analysis were used for survival analysis and the predictors.

**Results:**

A total of 155 patients admitted with RHD during the study period. Of these, 14 (9.0%) deaths were reported as RHD related mortality with the mean age ± standard deviation of 11.9 ± 2.9 years. Median follow up period was 16 months. The survival rate at 1, 4, and 8 years were 93%, 86.1% and 60%, respectively. Survival was lower in patients with severe valve lesions and NYHA functional class III-IV at the time of diagnosis. Multivariate Cox-regression analysis showed the significant predictors for mortality were poor adherence to penicillin and congestive heart failure, HR 12.7 (95%CI 1.4–113.5) and 18.6 (95%CI 1.8–185.0) respectively.

**Conclusion:**

Approximately, only six in 10 children are able to survive at eight years after diagnosis. Poor adherence to penicillin and congestive heart failure were predictors for death. This study emphasizes the needs to improve the adherence of children with RHD and early detection of subclinical carditis in children.

## Background

Acute rheumatic fever (ARF) and rheumatic heart disease (RHD) remain preventable cardiovascular deaths in the developing world and affect children and young adults in their productive age. Indonesia is an endemic country of RHD and becomes the fourth most prevalence country in the world (after China, India and Pakistan) with 1.18 million cases per year in 2015 [1,2].

The mortality rate of RHD in Indonesia was estimated around 4.8 per 100,000 population at risk (CI 95%, 4.4–5.1) [1,2,3]. This mortality rate was higher than mortality caused by malaria infection at the same year, 3.02 per 100,000 population at risk [4]. Global mortality rates have significantly reduced from 1990 to 2015 with the implementations of national program of prevention, control and eradication of ARF and RHD in many countries such as Australia, New Zealand, India and Africa Union [5,6,7,8]. However, in Indonesia, RHD becomes the neglected disease reflected by not having an existing Indonesian national program of RHD up to now. This can be due to the underreported cases in Indonesia including the mortality rates. A study at the tertiary hospital in the capital city of Indonesia in 1995 found that the survival rates in children with RHD at 10 years after diagnosis was 69.1% [9]. Compared to the economic and health care situation in 1995, Indonesia has undergone several improvements of socioeconomic status and the healthcare systems [10]. Several studies have proved that improvement in socioeconomic and healthcare systems could improve the outcome of RHD [11].

Therefore, we conducted a study to evaluate the outcome of RHD in Indonesian children particularly the survival rates and the predictors of mortality at Dr. Sardjito Hospital, a tertiary and teaching hospital in Yogyakarta, Indonesia.

## Methods

A retrospective cohort study using secondary data from RHD registry and medical records of Dr. Sardjito Hospital, Indonesia, was conducted from 2011–2021. All children (5–18 years) admitted with the diagnosis of RHD were recruited. RHD diagnosis were based on the 2012 World Heart Federation (WHF) RHD echocardiography criteria (Table 1) [12]. Patients were included if definite and borderline criteria were fulfilled. We excluded patients with incomplete medical records and proven as ARF without RHD after follow up visit (completely resolved valve lesions after management of ARF).

**Table 1:** 2012 WHF criteria for echocardiographic diagnosis of RHD for individuals aged ≤ 20 years.


Definite RHD (either A, B, C, or D)	Pathological MR and at least two morphologicalfeatures of RHD of the MV MS mean gradient ≥4mmHg* Pathological AR and at least two morphological features of RHD of the AV^‡^ Borderline disease of both the AVand MV^§^

Borderline RHD (either A, B, or C)	At least two morphological features of RHD of the MV without pathological MR or MS Pathological MR Pathological AR

Normal echocardiographic findings (all of A, B, C, and D)	MR that does not meet all four Doppler echocardiographic criteria (physiological MR) AR that does not meet all four Doppler echocardiographic criteria (physiological AR) An isolated morphological feature of RHD of the MV (for example, valvular thickening) without any associated pathological stenosis or regurgitation D. Morphological feature of RHD of the AV (for example, valvular thickening) without any associated pathological stenosis or regurgitation


*Congenital MV anomalies must be excluded. Furthermore, inflow obstruction due to nonrheumatic mitral annular calcification must be excluded in adults. ^‡^Bicuspid AV, dilated aortic root, and hypertension must be excluded. ^§^Combined AR and MR in high prevalence regions and in the absence of congenital heart disease is regarded as rheumatic. Abbreviations: AR, aortic regurgitation; AV, aortic valve; MR, mitral regurgitation; MS, mitral stenosis; MV, mitral valve; RHD, rheumatic heart disease; WHF, World Heart Federation.

### Data collection

At the first hospital admission, baseline characteristics and clinical predictors of mortality based on previous studies [13] were recorded (age at diagnosis (years), severe malnutrition, adherence to penicillin prophylaxis, congestive heart failure, pulmonary hypertension, atrial fibrillation, ejection fraction (EF), and left ventricle internal diameter at diastole (LVIDd)). Severe malnutrition was classified based on WHO growth chart at time of diagnosis (weight-for-height less than -3 SD or body mass index-for-age less than -3 SD).

After the first diagnosis of RHD, patients were managed according to local clinical guidelines for RHD. Then, they were scheduled for hospital visit for every 3–6 months. At each subsequent visit, the medical staffs asked patients to self-report their adherence to penicillin prophylaxis. Patients were classified as ‘adherent’ to secondary prophylaxis when they self-reported their adherence and consumed more than 80% of the prescribed oral penicillin for 12 months. ‘Non-adherent’ referred to the consumptions of oral penicillin less than 80% of the described doses based.

Congestive heart failure (CHF) was diagnosed if any two of the following three criterias were present: (1) symptoms (dyspnea on exertion or at rest, orthopnea, nocturnal paroxysmal dyspnea, or ankle edema) or signs (rales, increased jugular venous pressure, or ankle edema) of CHF; (2) radiological signs of pulmonary congestion; and (3) treatment with diuretics [13]. Atrial fibrillation was assessed based on electrocardiogram at the time of diagnosis.

### Echocardiography

All echocardiograms were performed by three experienced pediatric cardiologists with high interobserver agreement (intraclass correlation coefficient 0.89) using a Phillips Affinity 70G echocardiography. The pediatric cardiologists agreed to the definition of RHD based on 2012 World Heart Federation (WHF) RHD echocardiography criteria [12]. Two-dimensional and color images were used to asses valve severity based on American College of Cardiology/American Heart Association recommendations (Table 2) [14]. The inter-operator variability often occurred on the borderline of RHD due to the subjective assessment of mitral valve chordal thickening as morphological characteristics of RHD. Discrepancies also occurred in patients with congenital MR. In case of discrepancies, the pediatric cardiologists agreed to use the isolated pathological mitral regurgitation criteria to define the borderline RHD. The isolated pathological mitral regurgitation criteria are four times more likely to occur in high-risk population and the morphological features of RHD take time to develop [12].

**Table 2:** Valve severity based on American College of Cardiology/American Heart Association recommendations.


ECHOCARDIOGRAPHIC PARAMETERS	MITRAL REGURGITATION		

	MILD	MODERATE	SEVERE

Color Doppler jet area	Small central jet area <20% LA^1^ area	Central jet MR 20%–40% LA or late systolic eccentric jet MR	Central jet MR^2^ > 40% LA or holosystolic eccentric jet MR

Vena contracta width (cm)	Small vena contracta <0.3 cm	0.3–0.6 cm	≥0.7 cm

Regurgitant volume	Less than 30 ml	30–59 mL	≥60 mL

Regurgitant fraction	Less than 30%	<50%	≥50%

	**AORTIC REGURGITATION**		

	**MILD**	**MODERATE**	**SEVERE**

Jet width	<25% of LVOT^3^	25%-64% of LVOT	≥65% of LVOT

Vena contracta	<0.3 cm	0.3–0.6 cm	≥0.7 cm

Regurgitant volume	Less than 30 ml	30–59 mL	≥60 mL

Regurgitant fraction	Less than 30%	<50%	≥50%


^1^LA, Left atrium. ^2^MR, Mitral regurgitation. ^3^LVOT, Left ventricular outflow tract.

Pulmonary hypertension was determined using peak tricuspid regurgitation velocity >3.4 m/s in the absence of pulmonary outflow obstruction [15]. M-mode were used to obtain *left ventricle internal diameter at end-diastole* (LVIDd) and ejection fraction (EF). LVIDd (in millimeter) were converted to body surface area-adjusted z-scores for common M-Mode measurements [16]. All data were obtained from medical records and echocardiography registry.

### Outcome measures

After diagnosed with RHD, all patients were prescribed oral penicillin prophylaxis which could be taken at their nearest district hospitals. We scheduled echocardiography evaluations of all RHD patients for every six months at the out-patient clinics. Patients were followed up with until their deaths or last visit in our outpatient clinic. The outcome of interest was disease-related deaths (30-days mortality and hospital mortality). Deaths related to hemodynamic complication of RHD (arrhythmia, congestive heart failure, stroke, pulmonary embolism and pulmonary hypertension) were also included in the outcome of this study. Subjects were censored if they were alive until May 31, 2021 or lost to follow up. This study was approved without individual patients’ consents needed by the Ethics Committee of the Faculty of Medicine, Universitas Gadjah Mada, Yogyakarta, Indonesia (No. KE/FK/1380/EC/2020).

### Data analysis

Baseline characteristics and outcome were presented using descriptive statistics. Continuous variables were expressed as means with standard deviations or medians with interquartile ranges as appropriate and categorical variables as frequencies and percentages. Survival rates were assessed with Kaplan-Meier curve and log-rank test to compare the survival based on the severity of valve lesion and NYHA class at time of diagnosis. Age, severe malnutrition, atrial fibrillation, congestive heart failure, pulmonary hypertension, poor adherence to penicillin prophylaxis, low ejection fraction and the left ventricle internal diameter during diastole (LVIDd) were hypothesized mortality predictors and analyzed with univariate Cox proportional hazards models with 95% confidence interval (CI) and p value. Only the unadjusted hazard ratio (HR) with p value less than 0.05 were included in the multivariate Cox proportional hazards models for adjustment of HR.

Data was analyzed using R version 3.6.2 (2019–12–12) ‘Dark and Stormy Night’ Copyright (C) 2019 The R Foundation for Statistical Computing.

## Results

**Figure 1:**
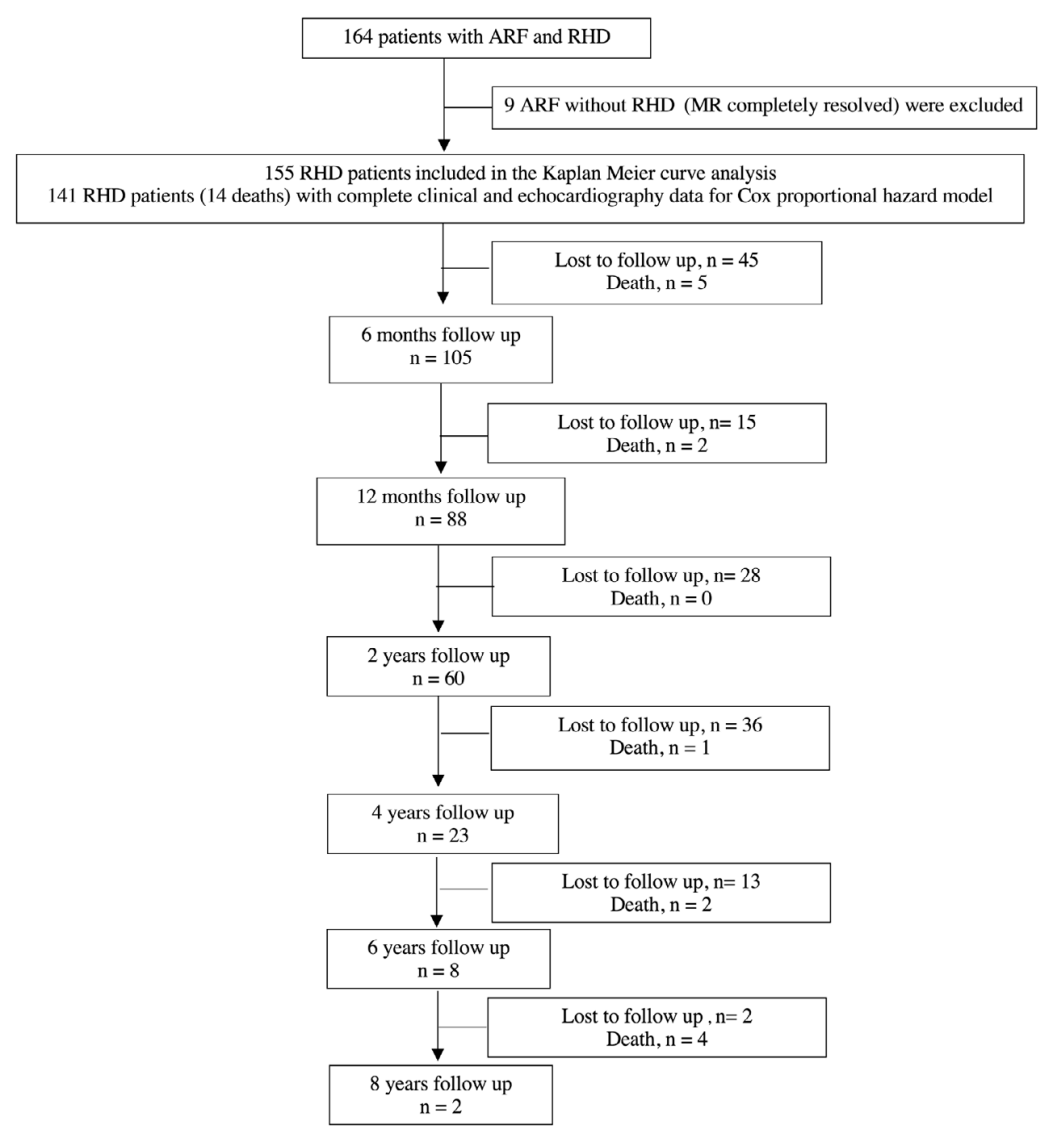
Study flow chart and follow up.

Baseline characteristics and flow of the study subjects are shown in Table 3 and Figure 1. Follow up duration ranged from 1 day to 8.5 years, with median follow up of 16 months. In over 10 years (2011–2021), there were 155 RHD children admitted to our hospital. Of these, 14 (9.0%) children died because of hemodynamic complications of RHD. The majority cause of deaths was severe heart failure which led to cardiogenic shock (Table 4). Mean age at diagnosis was 11.9 ± 2.9 years. The most common complication at initial presentation was pulmonary hypertension. More than half of these children (55.4%) presented with New York Heart Association (NYHA) functional class III and IV. Majority of these children (61.1%) already had severe valve lesions at initial presentations.

**Table 3:** Baseline characteristics of 155 children with RHD.


CHARACTERISTICS	SUBJECTS (n = 155)

Age in years, mean (SD)	11.9 (2.9)

Sex, n (%)	

– Male	90 (58.1)

– Female	65 (41.9)

Complications, n (%)	

– Infective endocarditis	2 (1.3)

– Atrial fibrillation	4 (2.6)

– Congestive heart failure	27 (17.4)

– Pericardial effusion	11 (7.1)

– Pneumonia	2 (1,3)

– *Total AV Block*	1 (0.6)

– Pulmonary hypertension	38 (24.5)

NYHA functional class at initial presentation, n (%)	

– NYHA I	29 (18.7)

– NYHA II	40 (25.8)

– NYHA III	64 (41.2)

– NYHA IV	22 (14.2)

Echocardiography parameter:	

– LVIDd, median (min-max)	51.2 (32.5–85.0)

– EF (%), median (min-max)	64.4 (15.0–82.0)

– Valve lesion severity, n (%)	

Severe	96 (61.1)

Moderate	34 (22.1)

Mild	25 (16.8)


AV, atrioventricular. LVIDd, Left Ventricular Internal Diameter end distole; EF, Ejection Fraction. NYHA, New York Heart Association.

**Table 4:** Cause of deaths and comorbidities of patients with RHD.


CAUSE OF DEATH	n (%)

Intracranial hemorrhage	1 (0.6)

Cardiogenic shock	12 (7.7)

Pulmonary hypertension crisis	1 (0.6)

**COMORBIDITY**	**n (%)**

Endocarditis	1 (0.6)

Thyroid malignancy	1 (0.6)

Pneumonia	4 (2.4)

Scabies	1 (0.6)

Total atrioventricular block	1 (0.6)


Mitral valves were the most common affected valves (98%) whether in combined valve lesions or isolated. Isolated aortic valve lesions occurred in three patients (3%). More than half of these children (71.6%) presented with multiple valves lesions at the time of first diagnosis (Table 5).

**Table 5:** Characteristics of valves lesions in children with RHD (n = 155).


VALVES LESIONS	n (%)

**a.** Single valve lesion	

Mitral valve:	

– Mitral regurgitation (MR)^a^	41 (27.5)

– Mitral stenosis (MS)^b^	0 (0.0)

Aortic valve	

– Aortic regurgitation (AR)^c^	3 (2.0)

– Aortic stenosis (AS)^d^	0 (0.0)

– Combination of AR^c^ and AS^d^	0 (0.0)

**b.** Multiple valve lesions	

– MR^a^ + AR^c^	41 (26.5)

– MR^a^ + Tricuspid regurgitation (TR)^e^	20 (12.9)

– MR^a^ + Pulmonal regurgitation^f^	2 (1.4)

– MR^a^ + MS^b^ +TR^e^	1 (0.7)

– MR^a^ + MS^b^ + AR^c^ + TR^e^	3 (2.0)

– MR^a^ + AR^c^ + TR^e^	21 (13.5)

– MR^a^ + TR^e^ + PR^f^	8 (5.4)

– MR^a^ + AR^c^ + PR^f^	1 (0.7)

– MR^a^ + AR^c^ + TR^e^ + PR^f^	14 (9.0)


Notes: ^a^MR, *Mitral Regurgitation*; ^b^MS, *Mitral Stenosis*; ^c^AR, *Aortic Regurgitation*; ^d^AS, *Aortic Stenosis*; ^e^TR, *Tricuspid Regurgitation*; ^f^PR, *Pulmonal Regurgitation*.

**Figure 2:**
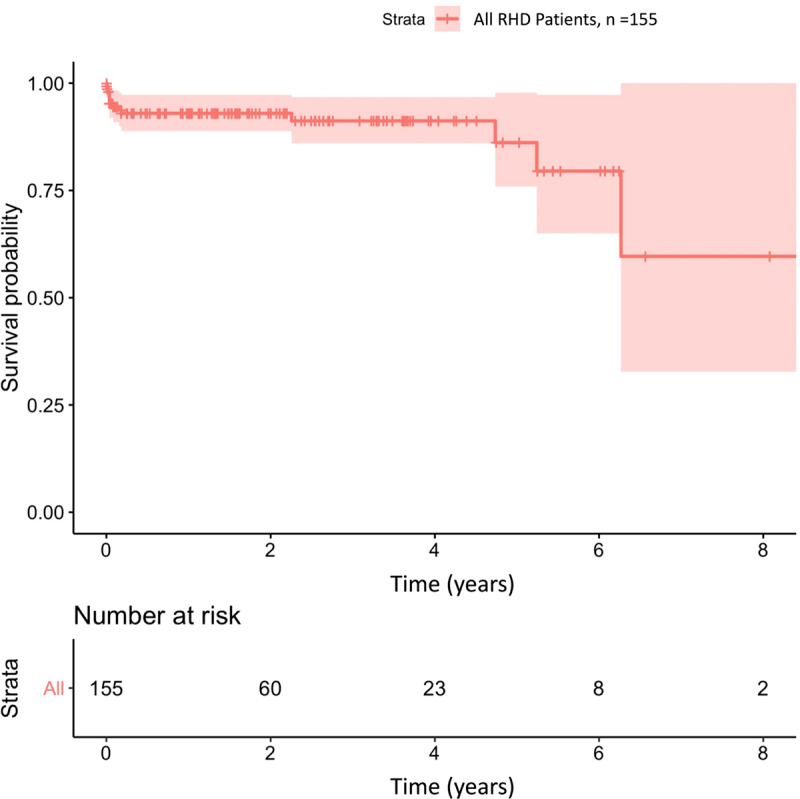
Survival analysis of children with RHD.

**Figure 3:**
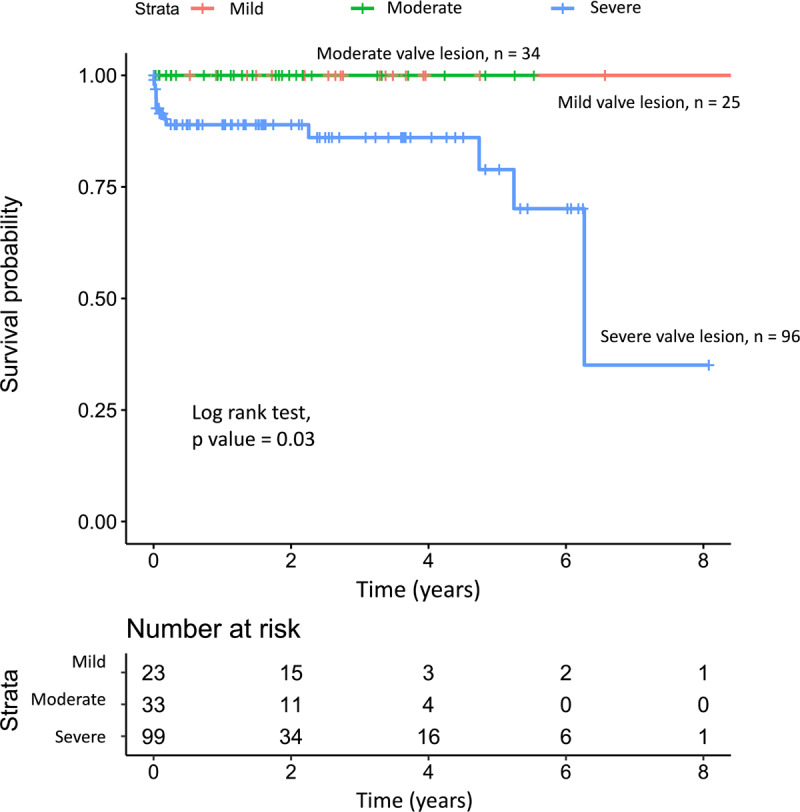
Kaplan-Meier curve stratified by valve severity.

Survival rates at 1, 4, and 8 years were 93.0%, 86.1% and 60% (Figure 2). Log rank test showed that children with severe valve lesions had lower survival rate than mild to moderate valve lesions (p = 0.014) (Figure 3).

**Figure 4:**
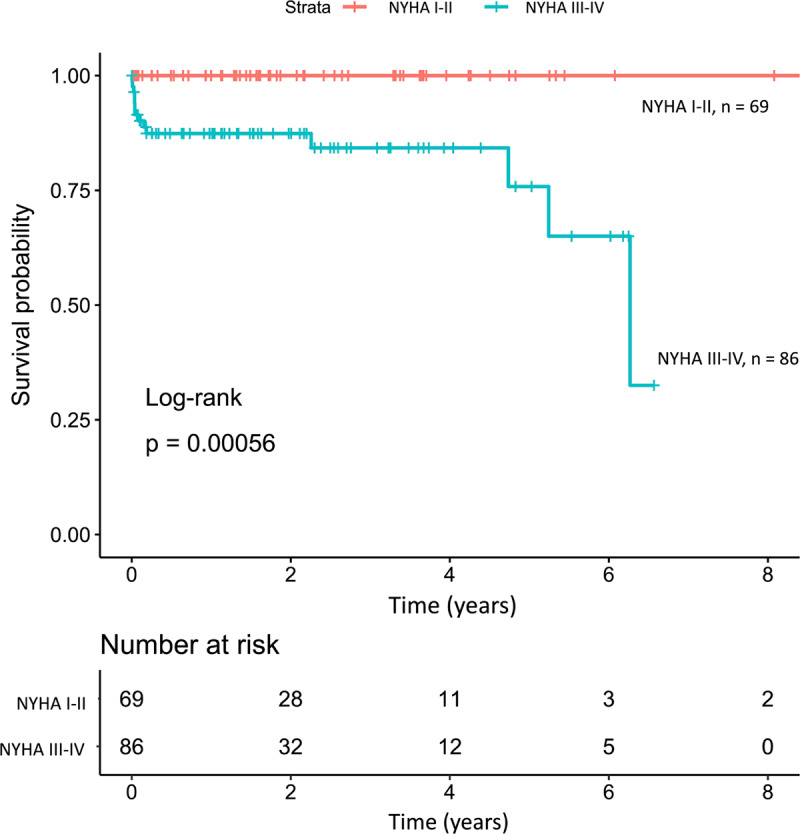
Kaplan-Meier curve stratified by NYHA functional class at first diagnosis.

In addition to the lower survival rates in the severe valves lesion groups, the survival of children with NYHA functional class III-IV was also lower, with median survival time of six years and two months while all children NYHA class I-II survived more than eight years after diagnosis (Figure 4).

Due to the incomplete clinical and echocardiography data of mortality predictors, Cox-proportional hazard model could only be conducted for 149 patients with nine events. Univariable Cox proportional hazard model showed that severe malnutrition, poor adherence to penicillin prophylaxis, congestive heart failure, pulmonary hypertension and LVIDd were predictors for RHD mortality (Table 6). Multivariable analysis showed that poor adherence to penicillin prophylaxis was an independent predictor for deaths (*adjusted HR* 14.3 [95%CI 1.3–156.6]). However, this analysis suffered from model instability as the number of events per variable less than 10. We did the secondary analysis by eliminating the competing determinants of congestive heart failure which were pulmonary hypertension and LVIDd. These factors can be considered as the intermediate factors before the congestive heart failure occurred and this assumption was proved by the statistically significant interaction of these three factors (p value of interaction < 0.05 by nested model for interaction term). We found the congestive heart failure and poor adherence were the predictors of deaths from the simplified Cox proportional hazard model (Table 7).

**Table 6:** Predictors of mortality in children with RHD with Cox Proportional Hazard Model.


PREDICTORS	DIED (n = 14)	ALIVE (n = 141)	UNADJUSTED HAZARD RATIO (CI 95%)	p	ADJUSTED HAZARD RATIO (CI 95%)	p

Age in years, mean (SD)	12.8 (2.8)	11.8 (2.9)	1.2 (0.9–1.5)	0.06		

Severe malnutrition, n (%)	8 (57.1)	13 (9.2)	7.3 (2.5–21.5)	<0.0001	1.6 (0.2–14.1)	0.95

Atrial fibrillation, n (%)	3 (21.4)	1 (0.7)	1.4 (0.8–22.3)	0.07		

Congestive heart failure, n (%)	10 (71.4)	16 (11.3)	11.5 (3.6–36.7)	<0.0001	8.9 (0.7–118.8)	0.09

Pulmonary hypertension, n (%)	11 (78.6)	28 (19.9)	10.1 (2.8–36.3)	<0.0001	0.9 (0.1–9.7)	0.98

Poor adherence to penicillin prophylaxis	8 (88.9)	40 (28.6)	34.6 (4.3–282.8)	<0.0001	14.3 (1.3–156.6)	0.02

EF < 55%, n (%)	4 (28.6)	16 (11.3)	2.9 (0.8–9.4)	0.07		

Z-scores LVIDd, mean (SD)	3.2 (2.1)	1.6 (1.9)	1.5 (1.1–2.1)	0.02	1.1 (0.6–2.3)	0.71


Severe malnutrition, Body mass index-per-age ≤ –3SD based on WHO 2006 growth chart. ‘Non-adherent’ or ‘poor adherent’ referred to the consumptions of oral penicillin were less than 80% of described doses based on patients’ self-reports for 12 months. LVIDd, *Left Ventricular Internal Diameter end distole* body surface area-adjusted z-scores EF, *Ejection Fraction*

**Table 7:** Simplified multivariable Cox Proportional Hazard Model of RHD mortality predictors.


PREDICTORS	ADJUSTED HAZARD RATIO (CI 95%)	p

Severe malnutrition, n (%)	0.6 (0.1–3.2)	0.51

Congestive heart failure, n (%)	18.6 (1.8–185.0)	0.01

Poor adherence to penicillin prophylaxis	12.7 (1.4–113.5)	0.02


## Discussion

In this present study, we used a single center experience at a tertiary cardiac center hospital in Indonesia. Compared to the data from 30 years ago at the same hospital, the total number of new cases per year decreased (15 cases per year vs 25 cases per year in 1980–1989) [17]. To our knowledge, this study was the second study in Indonesia that reported the survival rates of RHD in children in a tertiary hospital. Sastroasmoro et al. in 1995 found the survival rates at 10 years after diagnosis was 69.1% [9]. Our results were quite similar to the previous study.

Survival rates were lower in severe valve lesions in our study. A similar study in Africa showed that none of the patients without valve surgery could survive, with the youngest mortality being 25 years old [8]. A study in Australia showed that 10% of severe RHD died within six years after diagnosis without surgical intervention [18]. Research in North India also showed that 58% patients with severe valves lesions died within one year after diagnosis [19]. Severe valve lesions were independent predictors for deaths with *Hazard Ratio (HR)* 2.36 (95%CI 1.80–3.11) in Asia-Africa multicenter studies [20].

Our study also showed that children with NYHA functional class III and IV at initial diagnosis had higher mortality rate. This result was similar to a study from 14 low-and middle-income countries that showed the NYHA functional class was a predictor of mortality with HR 1.67 (95%CI 1.32–2.10) [20]. Severe valve lesion and lower functional class at initial presentations were conditions that reflect the delayed diagnosis of RHD in Indonesian children.

Even though our survival was not different than a study from 25 years ago, the cumulative mortality rates were lower than that the previous (18.3% vs 9.0%). Studies from high-income countries stated that RHD ceased to be a public health problem several decades ago due to the improvement of socioeconomic status [1]. The reduction of this mortality rates probably related to the improvements of healthcare system and socioeconomic status in Indonesia. However, this result could not be generalized without further considerations due to the different hospital conditions.

Mitral valve lesion was the majority valve lesions in this study which was similar to studies from many countries (India, Australia and Europe) [18,19,20]. Mitral valve lesions were started by circulating autoantibody that binds to the surface endothelium of mitral valve and enhances the vascular cell adhesion protein 1 expression. The activated endothelium will facilitate T-lymphocyte to infiltrate the sub endothelium of the valve and lead to inflammation. The inflammation will expose the extracellular matrix which make the damage of the valves become worse [21]. Mitral regurgitation will occur two decades earlier that mitral stenosis and become the most common valve lesion in children under 18 years old [18].

Multivariable Cox proportional analysis showed that poor adherence to Penicillin prophylaxis was a predictor of deaths. Several longitudinal studies in many countries also showed that penicillin prophylaxis could reduce the progressivity of RHD and lowered the mortality rates [22,23,24,25]. The risk of mortality in patients with poor adherence to penicillin injection as prophylaxis was 3.81 times higher than patients with good adherence (HR 3.81, 95%CI 1.92–7.63, p = 0,001) [26].

Congestive heart failure can occur due to the hemodynamic complication of recurrent carditis and residual valves lesion after ARF resolved. The first prospective study by Bland and Jones in 1951 revealed that from 20 years period of observation, 80% of RHD mortality occurred in patients with congestive heart [27]. Congestive heart failure also became a predictor of RHD mortality in Uganda with HR 8.36 (95%CI 3.2–21.3) [26]. This study showed the same results with increase mortality risk by 18.6 times.

Severe malnutrition, pulmonary hypertension and left ventricle dilatation were statistically significant with univariate analysis and became insignificant in multivariate analysis. The possible cause could be the effect of low event rate and the presence of interactions between these predictors as all of them were the hemodynamic complications of RHD.

Malnutrition played a role in increasing mortality risk from the univariable analysis with unadjusted HR 8.7 (95%CI 2.8–27.0). This result was in agreement to a study of heart failure in children in which the 18-month-survival rates of congestive heart failure children with severe malnutrition 23% (95%CI 0–46). Congestive heart failure will lead to low nutritional intake due to the intestinal edema, anorexia and malabsorption. On the contrary, congestive heart failure will increase the energy expenditures. This process will deteriorate the nutritional status and lead to severe malnutrition. The combination of congestive heart failure and malnutrition will increase the right ventricle load and reduce the exercise capacity and lead to premature deaths [28].

Pulmonary hypertension increased the risk of mortality by 10.1 times (95%CI 2.8–36.3). Patients who already had pulmonary hypertension should undergo surgical valve repair as soon as possible. A previous study has proved that lower survival rates in RHD patients with pulmonary hypertension [29].

Left ventricle dilatation was the predictor for mortality in Uganda and similar to this study result with univariable analysis [26]. Chronic mitral regurgitation will increase the volume load of the left atrium and left ventricle and the dilatation of left ventricle more than 55 mm during diastole was a predictor of mortality. However, this cutoff value was derived from adults. Measurement of left ventricle dilatation in children should be adjusted with body surface area. As far as we are aware, there was no study of RHD which compares the *z-scores of* LVIDd to the mortality. However, higher *z-scores* of LVIDd at initial presentation have been proven as a predictor of deaths in myocarditis in children [30].

Although age has been proven as predictor for deaths in other studies, our study could not prove this, probably due to the limited inclusion criteria. As we only involved children, we did not have a comparison of survival in adults. Left ventricle disfunction was not a predictor of death in children with RHD. Left ventricle can maintain the systolic function for the long period and the disfunction will occur in adults [31].

Atrial fibrillation also did not correlate with mortality in children with RHD. This result was consistent with the pathophysiology of atrial fibrillation in RHD. Atrial fibrillation occurs in mitral stenosis in which the mitral commissures have fused, thickened and calcified. This leads to enlargement of left atrium and stimulates abnormal atrial tissue substrates which rapidly fire ectopic foci [32].

This study has some limitations. Since our study was a relatively small cohort study with a corresponding low event rate, the multivariable Cox proportional hazard modeling suffered from model instability. Therefore, we did the simplified analysis of Cox proportional hazard model by eliminating the competing determinants. Another limitation was the use of secondary data, which could overestimate the survival for the total Indonesian children with RHD and the possibilities of deaths prior to hospital care. The subjective measurements of adherence also potentiate the subjective bias of this study. Furthermore, Indonesia has widely implemented a National Health Insurance Program which requires our patients to receive their monthly medication at the nearest hospitals of their domicile especially after 12 months of diagnosis. Thus, we could only measure the adherence for 12 months after diagnosis.

## Conclusion

Approximately, only six in 10 children will be able to survive up to eight years after diagnosis. Poor adherence to penicillin and congestive heart failure are predictors for death. This study emphasizes the needs to improve the adherence of children with RHD and early detection of subclinical carditis in children.

## Additional File

The additional file for this article can be found as follows:

10.5334/gh.1160.s1 Dataset. RHD Data Set Indonesia Children for GH.
